# Individualized QT interval (QTi) is a powerful diagnostic tool in long QT syndrome: results from a large validation study

**DOI:** 10.3389/fcvm.2023.1097468

**Published:** 2023-05-11

**Authors:** Tomas Robyns, Dieter Nuyens, Bert Vandenberk, Peter Haemers, Jeroen Breckpot, Christophe Garweg, Joris Ector, Rik Willems

**Affiliations:** ^1^ Department of Cardiovascular Diseases, University Hospitals Leuven, Leuven, Belgium; ^2^ Department of Cardiovascular Sciences, University of Leuven, Leuven, Belgium; ^3^ Department of Cardiology, Ziekenhuis Oost Limburg Genk, Genk, Belgium; ^4^ Department of Human Genetics, University Hospitals Leuven, Leuven, Belgium

**Keywords:** individualized QT correction, QTI, long QT syndrome, holter, LQTS

## Abstract

**Aims:**

Diagnosis of Long QT syndrome (LQTS) is based on prolongation of the QT interval corrected for heart rate (QTc) on surface ECG and genotyping. However, up to 25% of genotype positive patients have a normal QTc interval. We recently showed that individualized QT interval (QTi) derived from 24 h holter data and defined as the QT value at the intersection of an RR interval of 1,000 ms with the linear regression line fitted through QT-RR data points of each individual patient was superior over QTc to predict mutation status in LQTS families. This study aimed to confirm the diagnostic value of QTi, fine-tune its cut-off value and evaluate intra-individual variability in patients with LQTS.

**Methods:**

From the Telemetric and Holter ECG Warehouse, 201 recordings from control individuals and 393 recordings from 254 LQTS patients were analysed. Cut-off values were obtained from ROC curves and validated against an in house LQTS and control cohort.

**Results:**

ROC curves indicated very good discrimination between controls and LQTS patients with QTi, both in females (AUC 0.96) and males (AUC 0.97). Using a gender dependent cut-off of 445 ms in females and 430 ms in males, a sensitivity of 88% and specificity of 96% were achieved, which was confirmed in the validation cohort. No significant intra-individual variability in QTi was observed in 76 LQTS patients for whom at least two holter recordings were available (483 ± 36 ms vs. 489 ± 42 ms, *p* = 0.11).

**Conclusions:**

This study confirms our initial findings and supports the use of QTi in the evaluation of LQTS families. Using the novel gender dependent cut-off values, a high diagnostic accuracy was achieved.

## Introduction

**Figure 1:**
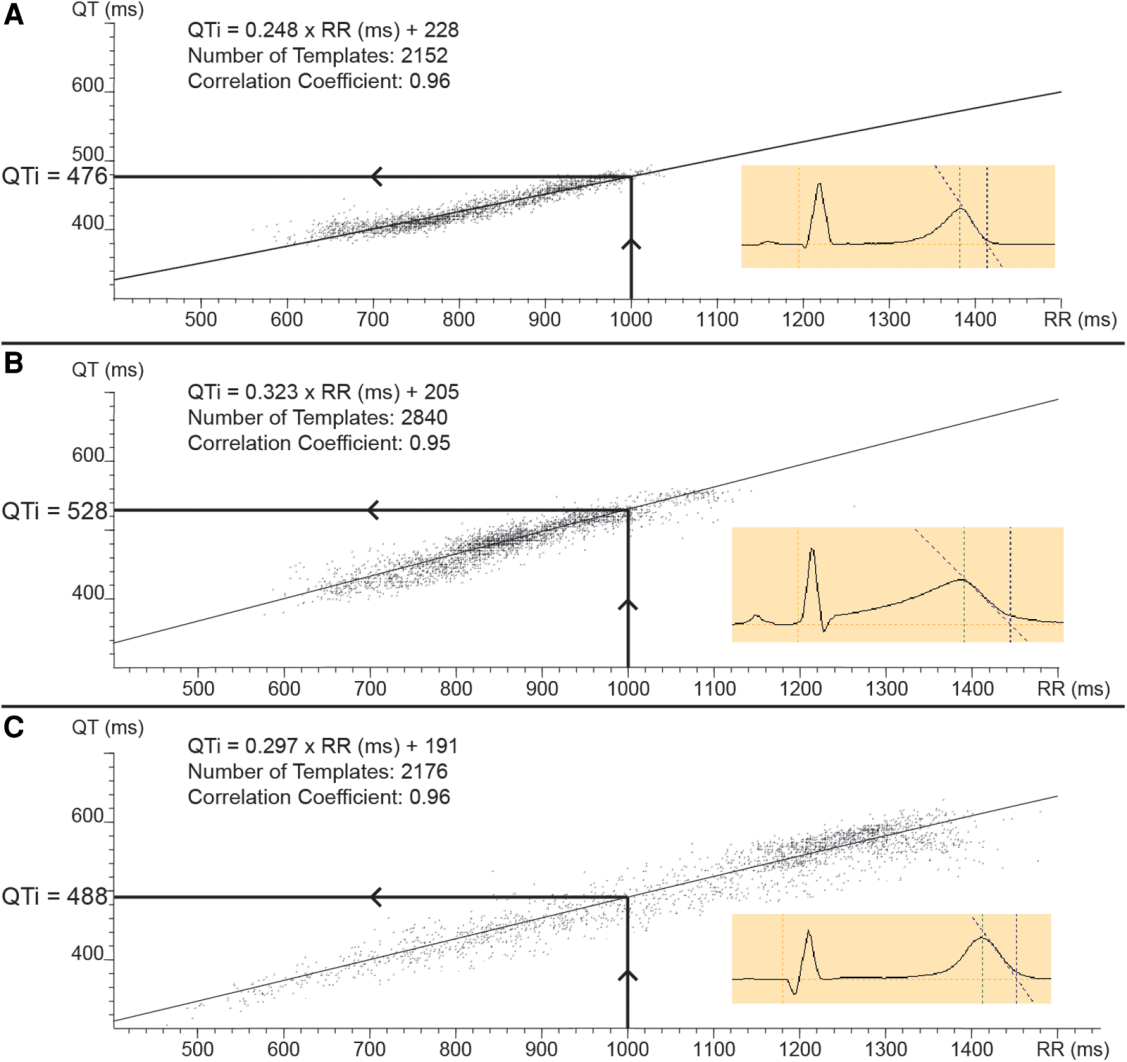
Illustration of the methodology of individualized QT correction (QTi). Templates of 30 s of ECG of a 24 h holter recording are plotted on a QT-RR data plot (figure insets show examples of such a template). Linear regression of this data set results in an individual correction formula (QTi = α × RR + β). QTi was defined as the QT value at the intersection of an RR interval of 1,000 ms with the linear regression curve, as is illustrated in the different panels by the arrows. (Panel **A**) shows a plot of a LQT1 patient with a borderline increased QT rate dependence (factor α). (Panel **B**) illustrates a plot of a LQT2 patient with increased QT rate dependence. (Panel **C**) shows a plot of a LQT3 patient with increased QT rate dependence and a large distribution of data points across the spectrum of RR intervals.

Long QT syndrome (LQTS) is a genetic electrical heart disease associated with an increased risk of ventricular arrhythmia and sudden death (1). The disease is characterized by increased action potential duration due to changes in ion currents of which a decrease in repolarizing potassium current or an increase in inward sodium current are the main examples. This increase in action potential duration is reflected by an increase in the QT interval on the surface ECG. However, about 25% of LQTS mutation carriers appear to have a normal QT interval corrected for heart rate (QTc) on their resting ECG (2). Therefore, methods have been developed to unmask these concealed LQTS patients, like the effect of adrenergic stimulation and withdrawal on the QT interval during adrenaline infusion, exercise or brisk standing (3–5). However, other issues in the measurement of the QT interval (6), its circadian variability (7) and artificial QT correction for heart rate (8) all play a role in misdiagnosis of the syndrome (9). In acquired long QT syndrome, the superiority of an individualized QT correction using multiple ECG recordings over generalized QT correction formulae has been established in the past decade (8, 10). To overcome the aforementioned issues with the QTc interval and in analogy with individualized QT correction in drug induced LQTS, we recently evaluated individualized QT interval (QTi) in congenital LQTS. QTi is based upon the patients’ own QT rate dependence derived from 24 h holter recordings and eliminates the need for QT correction (11). QTi was defined as the QT interval at the intersection of an RR interval of 1,000 ms with the linear regression line fitted through QT-RR data points of each individual patient (Figure 1). We showed that QTi with a gender independent cut-off of 445 ms was superior over QT corrected for heart rate by Bazett's formula from a standard 12 lead ECG to identify mutation carriers in families with LQTS. However, these data were derived from a relatively small LQTS cohort (*N* = 69) necessitating confirmation in a larger cohort. Therefore, we performed this follow-up study to ratify our findings in an independent cohort, to finetune the cut-off value of QTi and to evaluate whether QT measured at different RR intervals (than 1,000 ms) would have any additional diagnostic value.

## Methods

### Patient selection

Holter recordings were obtained from the Telemetric and Holter ECG Warehouse (THEW) of the university of Rochester, NY (12). The first database (E-HOL-03-0480-013) is a French LQTS database. The database consists of 480 two or three channel 24 h holter recordings from 307 individual LQTS patients with Jervell Lange Nielsen syndrome (JLNS), long QT syndrome type 1 (LQT1), long QT syndrome type 2 (LQT2) or long QT syndrome type 3 (LQT3). Some limited phenotypical data is available including age, gender, symptomatology, treatment and the specific mutation. Since this database dates back from the beginning era of genotyping for LQTS, all genetic variants were re-assessed using the recent ACMG-AMP criteria (13). This was mainly done to prevent mislabelling of individuals with a benign genetic variant as definite LQTS based solely on genetic information. Only holter recordings from patients with a pathogenic or likely pathogenic variant were retained. Holter recordings from patients with JLNS or from patients carrying compound mutations were excluded because of obvious QT prolongation. Mutations were categorized according to their effect on the amino acid sequence and according to their topological location (details in Supplementary Data).

The second database (E-HOL-03-0202-003) consists of three channel 24 h holter recordings from 202 healthy individuals from the Intercity Digital Electrocardiogram Alliance (IDEAL). Strict criteria had to be fulfilled in order to be eligible for this study including no history of cardiovascular or other chronic disorders, normal clinical examination, normal resting ECG and no drug therapy.

Finally, novel cut-off values for QTi were validated in our previously described in-house cohort of LQTS patients (11).

### Holter measurements

If multiple holter recordings were available per patient, the first recording was chosen for the main analysis and the second holter was chosen for evaluation of intra-individual variability. The measurements were performed blinded for patient status. A detailed description of the methodology of the measurement of QTi was published previously (11). In short, the holter recordings were analysed by means of the commercially available QT analysis module in “Synescope” Holter software (Microport, Shanghai, China). The 24 h recordings were converted into 2,880 mean complex waveforms (templates) obtained at 30 s intervals. Based on the mean waveform, the software calculates the end of the T wave by determining the intersection between the maximum decreasing tangent of the final upslope or downslope of the T-wave and the isoelectric line (defined as voltage at QRS onset). The QT intervals (Y axis) were plotted against the mean RR intervals of the preceding 30 s of ECG (X axis). Subsequently linear regression of the QT-RR scatterplot was automatically performed by the software to create an individualized QT correction formula (QTi = α × RR + β) where α denotes a factor known as QT-RR slope that reflects QT rate dependence. QTi was defined as the QT interval obtained at the intersection with the linear regression line at an RR interval of 1,000 ms (Figure 1). A Pearson correlation coefficient of the linear regression was automatically calculated. Recordings with less than 4 h of useful registration were excluded.

### Statistics

All continuous variables are expressed as mean ± standard deviation and categorical variables as number (percentage). LQTS patients and control individuals were compared using two-sided Student *t*-test for continuous variables and chi square test for categorical variables. Comparison of continuous variables between multiple groups was done using one-way ANOVA with Tukey's *post hoc* testing. ROC curves were constructed to determine ideal cut-off values in males and females, to evaluate the performance of QTi at different RR intervals and to evaluate a cut-off for QT-RR slope. Comparison between ROC curves was done with the method of DeLong in MedCalc 12.7 (MedCalc Software, Ostend, Belgium). Comparison of QTi and QT-RR slope in patients with a repeat holter was done with a paired T-test. A *p*-value below 0.05 was considered statistically significant. Data were analysed with SPSS 28.0 (SPSS Inc., Chicago, Illinois, USA). Graphics were created with SPSS 28.0 or Graphpad Prism 6.0 (Graphpad, San Diego, California, USA).

## Results

### Patient cohort

**Figure 2:**
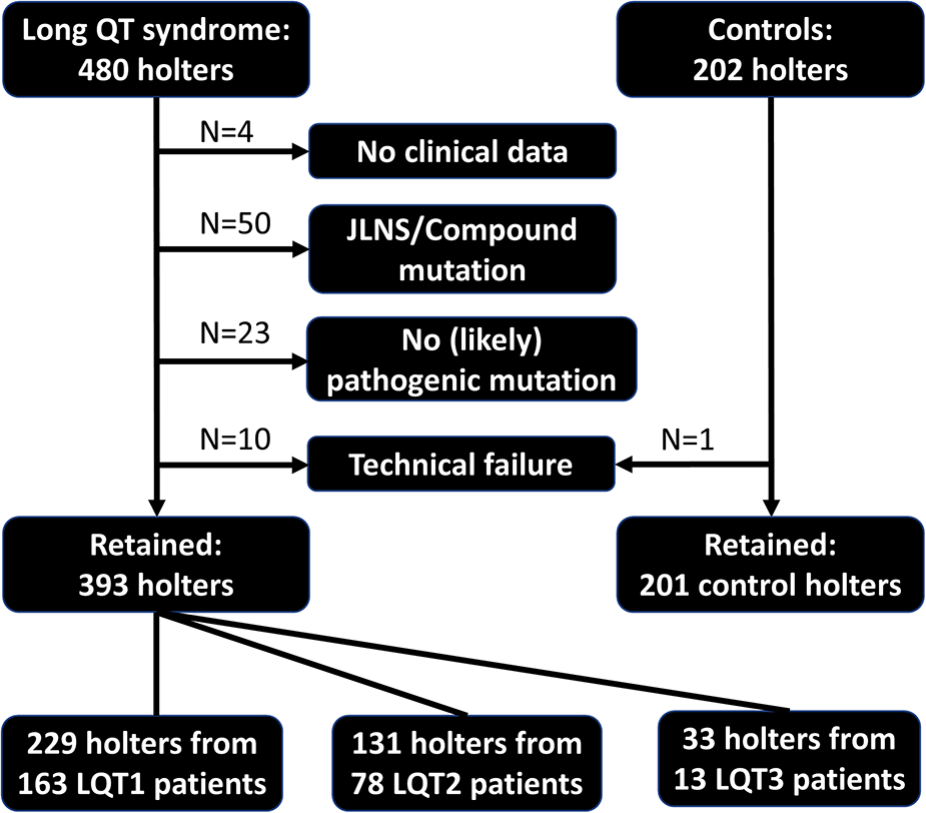
Study overview. Flowchart illustrating reasons for excluding holter recordings, total number of included holter recordings per LQTS subtype and control individuals. JLNS, Jervell Lange Nielsen syndrome.

In total, 682 24 h holter recordings were available from the 2 consulted databases. The flowchart in Figure 2 illustrates reasons for excluding holter recordings and final numbers of recordings per LQTS subtype and controls. The analysis could not be performed in only 11 out of 605 eligible recordings (1.8%). Reasons for this were absence of T waves in 4 recordings probably due to faulty digitalization of the signal, wrong QRS annotation in 3 recordings, extremely low T wave amplitude in 2 recordings and extreme QT prolongation in all templates in 2 recordings (QT > 700 ms is automatically filtered out by the software). Detailed information on the different mutations is summarized in Supplementary Material Table S1. Control patients were older compared to LQTS patients, while gender was similar (Table 1). Syncope was frequent in LQTS patients (37%), but not different between the LQTS subgroups (Table 2). Ventricular arrhythmia like torsades de pointes (TdP) and cardiac arrest only occurred in 5 (2%) LQTS patients.

**Table 1:** Demographic characteristics and holter variables in controls and LQTS patients.

	Controls	LQTS	*p*-value
Number	201	254	
Age	38 ± 16	28 ± 19	<0.001
Males	102 (51%)	115 (45%)	0.25
Syncope	0	93 (37%)	
CA/TdP	0	5 (2%)	
BB	2 (1%)	77 (30%)	<0.001
Templates	2,537 ± 363	2,101 ± 492	<0.001
RR mean (ms)	828 ± 110	883 ± 142	<0.001
QT-RR slope	0.168 ± 0.045	0.216 ± 0.090	<0.001
Correlation Coefficient	0.897 ± 0.083	0.787 ± 0.185	<0.001
QTi (ms)	399 ± 22	470 ± 35	<0.001

CA, cardiac arrest; TdP, torsades de Pointes; BB, beta blocker therapy; correlation coefficient, correlation coefficient of the linear regression of the QT-RR relationship; QTi, Individualized QT correction.

**Table 2:** Demographic characteristics and holter variables in LQTS subtypes.

	LQT1	LQT2	LQT3	*p*-value
Number	163	78	13	
Age	30 ± 19	25 ± 18	23 ± 18	0.101
Males	69 (42%)	39 (50%)	7 (54%)	0.436
Syncope	64 (39%)	26 (33%)	3 (23%)	0.390
CA/TdP	2 (1.2%)	3 (4%)	0	0.341
BB	43 (26%)	29 (37%)	5 (39%)	0.188
Templates	2,154 ± 439	2,034 ± 541	1,834 ± 685	0.026
RR mean (ms)	884 ± 135	882 ± 161	880 ± 116	0.995
QT-RR slope	0.180 ± 0.073	0.276 ± 0.083	0.315 ± 0.069	<0.001*^,^†
Correlation Coefficient	0.771 ± 0.203	0.803 ± 0.151	0.891 ± 0.048	0.041†
QTi (ms)	460 ± 31	486 ± 35	497 ± 44	<0.001*^,^†

CA, cardiac arrest; TdP, torsades de Pointes; BB, beta blocker therapy; correlation coefficient, correlation coefficient of the linear regression of the QT-RR relationship; QTi, individualized QT correction; LQT1, long QT type 1; LQT2, long QT type 2; LQT3, long QT type 3.

* LQT1 vs. LQT2.

^†^ LQT1 vs. LQT3.

### Cut off value for QTi and test performance

**Figure 3:**
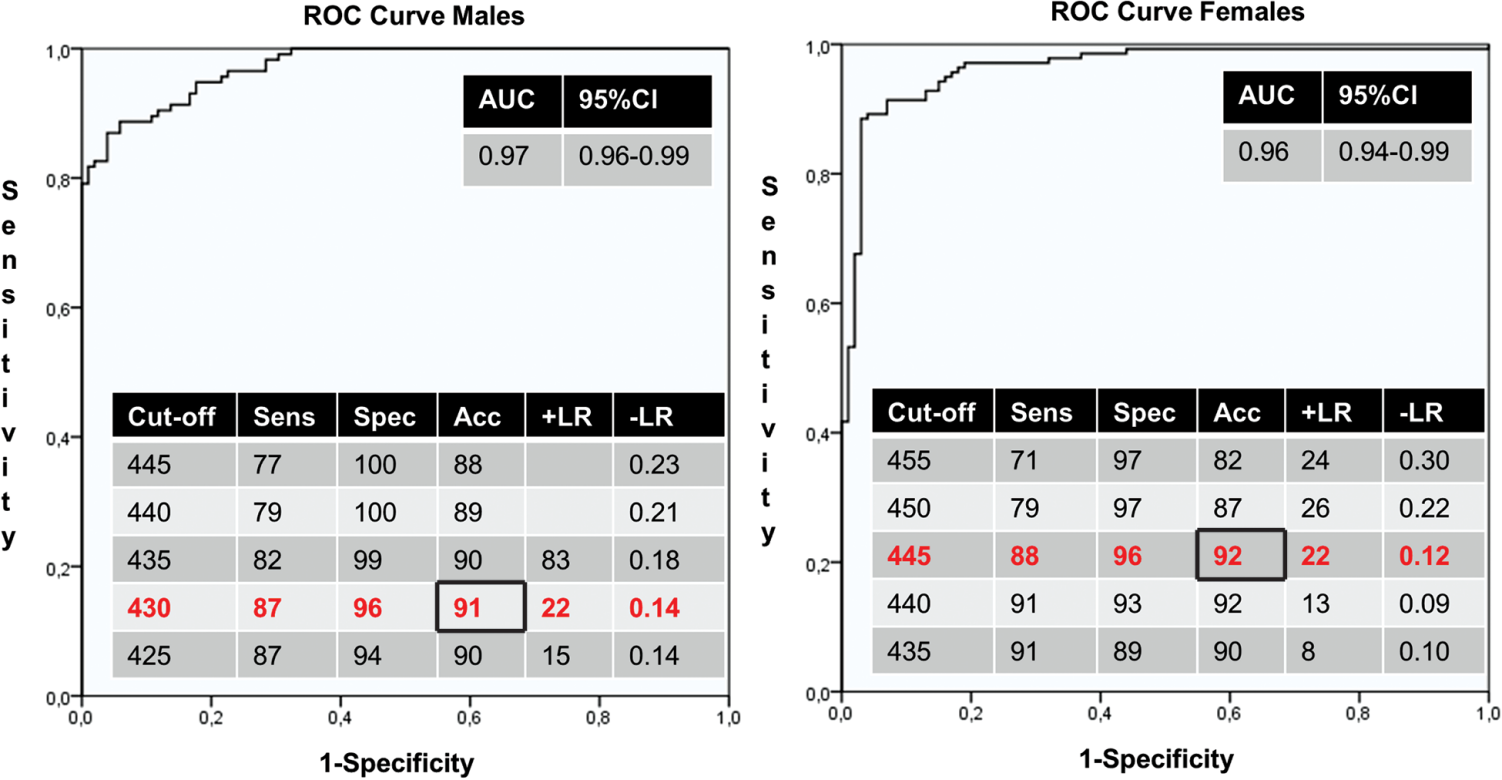
Receiver operator characteristics (ROC) curves of males (left panel) and females (right panel). The area under the curve (AUC) and diagnostic test results with different cut-offs are shown in the tables. 95% CI = 95% confidence interval; Sens, Sensitivity; Spec, Specificity; Acc, Accuracy; +LR, positive likelihood ratio; −LR, negative likelihood ratio.

The main results of the holter measurements of control individuals and LQTS patients according to gender are summarized in Table 3. The mean QTi in control individuals between males (*N* = 102) and females (*N* = 99) differed 19 ± 3 ms (390 ± 19 ms vs. 409 ± 21 ms, *p < *0.001), demonstrating that a gender dependent cut-off is preferred. The ROC curves indicated that the optimal cut-off value for QTi was 430 ms in males and 445 ms in females (Figure 3). Using these cut-off criteria in the total population, a sensitivity of 88%, specificity of 96%, diagnostic accuracy of 91%, positive likelihood ratio of 22 and negative likelihood ratio of 0.13 were achieved. Compared to these cut-offs, using the gender independent cut-off of 445 ms, a sensitivity of 83%, specificity of 98%, diagnostic accuracy of 90%, positive likelihood ration of 42 and negative likelihood ratio of 0.17 were achieved.

**Table 3:** LQTS vs. controls in males and females.

	Males			Females		
	Controls	LQTS	*p*	Controls	LQTS	*p*
Number	102	115		99	139	
Templates	2,571 ± 351	2,151 ± 477	<0.001	2,503 ± 374	2,059 ± 501	<0.001
RR (ms)	845 ± 105	906 ± 146	0.001	810 ± 113	864 ± 137	0.001
Corr Coeff	0.892 ± 0.091	0.807 ± 0.162	<0.001	0.903 ± 0.073	0.771 ± 0.201	<0.001
QT-RR slope	0.147 ± 0.031	0.212 ± 0.093	<0.001	0.189 ± 0.048	0.220 ± 0.088	0.002
QTi (ms)	390 ± 19	465 ± 36	<0.001	409 ± 21	475 ± 34	<0.001

Corr Coeff,  correlation coefficient of the linear regression of the QT—RR relationship; QTi, individualized QT correction; LQTS, long QT syndrome.

### QTi at different RR intervals

We evaluated whether assessment of individualized corrected QT interval at a different RR interval is preferred over QTi at 1,000 ms. Therefore, QT at an RR interval of 600, 700, 800, 900, 1,100, 1,200, 1,300 and 1,400 ms was calculated using the patients’ specific QT-RR linear regression formula (Supplementary Material Table S2). The ROC curves obtained using these QT values were compared with the standard QTi at an RR interval of 1,000 ms. Both in males and females, none of the QTi values resulted in a significant better AUC compared to conventional QTi at an RR interval of 1,000 ms (Supplementary Material Figure S1). In LQT1, QTi measured at 900 ms in both genders (*p = *0.01 in females and males) and 800 ms in males (*p = *0.046) had a significantly higher AUC compared to QTi at 1,000 ms (Supplementary Material Figure S2). In contrast, in LQT2 none of the QTi values measured at a different RR interval resulted in a higher AUC compared to QTi at 1,000 ms in both genders (Supplementary Material Figure S3). QTi measured at RR interval of 600 ms was significantly longer in LQT1 compared to LQT2, while QTi measured at 900 ms or at slower heart rates was longer in LQT2 compared to LQT1. Finally, 16.1% of LQTS patients and 16.4% of control individuals did not reach a maximum RR interval of 1,000 ms during their 24 h holter recording. There was no difference in QTi between patients who reached this threshold and those who did not in both the LQTS and control groups.

### Comparison of genotypes

QTi was more prolonged in LQT2 and LQT3 compared to LQT1 (Table 2). The test results for the 3 different genotypes are summarized in Table 4. In LQT1, the sensitivity was lower (85%) compared to LQT2 (94%) and LQT3 (92%). Therefore, we evaluated whether the degree of functional defect in LQT1 mutations influenced QT prolongation. QTi in 76 patients carrying a mutation with a dominant negative effect on I_Ks_ function was comparable to QTi from 47 patients carrying a non-dominant negative mutation (464 ± 31 ms vs. 457 ± 31 ms; *p = *0.25). Since only 4 patients with a mutation causing haplo-insufficiency and 4 with a mutation causing trafficking deficiency were included, no separate analysis was performed with these functional defects. No differences in QTi dependent on the type of mutation or topological location of the mutation were identified (Supplementary Material Table S3).

**Table 4:** Test results in different genotypes.

	AUC males	AUC females	Sensitivity	Specificity	+LR	−LR
LQT1	0.96 (0.94–0.99)	0.96 (0.93–0.99)	85%	96%	21.27	0.16
LQT2	0.99 (0.98–1)	0.98 (0.95–1)	94%	96%	23.51	0.07
LQT3	0.99 (0.98–1)	0.97 (0.91–1)	92%	96%	23.19	0.08

LQT1, long QT type 1; LQT2, long QT type 2; LQT3, long QT type 3; AUC, area under the curve; +LR, positive likelihood ratio; −LR, negative likelihood ratio.

### Intra-individual variability

In 76 LQTS patients, at least one repeat holter was available. There was no difference in QTi (483 ± 36 ms vs. 489 ± 42 ms, *p = *0.11) and QT-RR slope (0.234 ± 0.092 vs. 0.245 ± 0.104, *p = *0.27) between first and second measurement respectively. Using the proposed cut-off values, the sensitivity of the test was 92% using the first holter recording and 90% using the second holter recording. There was an increase of patients treated with beta blockers from 39% at first holter recording to 79% at follow-up holter recording. In 34 patients who were started on beta blocker therapy, there was no effect upon QTi (483 ± 28 vs. 487 ± 37, *p = *0.55) or QT-RR slope (0.201 ± 0.077 vs. 0.210 ± 0.086; *p = *0.62).

### QT rate dependence

**Figure 4:**
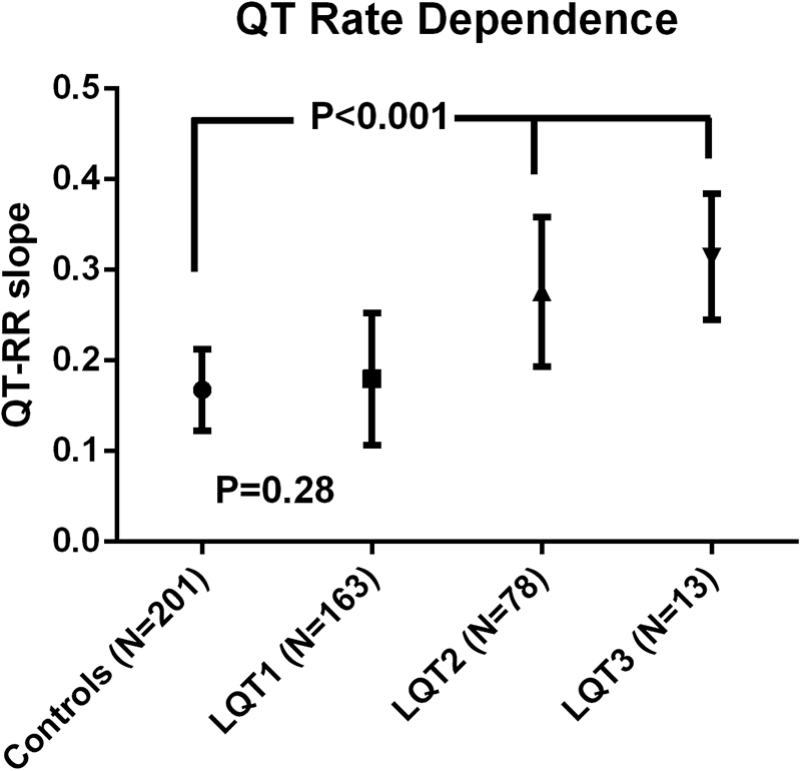
Qt rate dependence in LQTS subtypes. QT rate dependence was increased in LQT2 and LQT3 patients compared to LQT1 patients and control individuals. QT rate dependence was similar in LQT1 and controls.

In control individuals, QT rate dependence was higher in females compared to males (0.189 ± 0.048 vs. 0.147 ± 0.031; *p < *0.001). QT rate dependence was similar in LQT1 and controls, while it was increased in LQT2 and LQT3 (Figure 4). In LQT1, QT-RR slope remained significantly higher in females compared to males (0.193 ± 0.079 vs. 0.162 ± 0.059; *p = *0.006). This was not the case in LQT2, where the QT rate dependence was similar in females and males (0.273 ± 0.083 vs. 0.279 ± 0.083; *p = *0.74). There was no difference according to type of mutation (missense, truncating or splice site) in LQT1 and LQT2. In LQT1, C-loop mutations had a lower QT-RR slope compared to mutations located in the C-terminus (Supplementary Material Table S3). In LQT2, there was no difference dependent upon the location of the mutation. ROC analysis showed that QT-RR slope values ≥ 0.25 had a specificity of 95% (sensitivity 31%) to predict LQTS while a slope ≥ 0.30 had a specificity of 99% (sensitivity 16%).

### QTi novel cut-offs in validation cohort

Finally, we evaluated the novel proposed cut-off values of QTi in the cohort we described previously including 69 LQTS patients and 55 controls and used that cohort as a validation cohort (11). This resulted in only one reclassification of a male control patient with a false positive result using these novel cut-offs. In this validation cohort the diagnostic test results showed a sensitivity of 90%, specificity of 93%, positive likelihood ratio of 12, negative likelihood ratio of 0.11 and diagnostic accuracy of 91%. These numbers are very similar to those obtained in the derivation cohort here described.

### QTi in risk stratification for sudden death

Low numbers of patients with cardiac arrest or documented TdP precluded an analysis to identify risk markers for cardiac arrhythmia in this cohort. However, in symptomatic patients composed of patients with cardiac arrest, TdP and syncope (*N* = 98) QTi was significantly more prolonged compared to asymptomatic LQTS patients (480 ± 36 ms vs. 464 ± 34 ms; *p < *0.001). There was no difference between both groups regarding QT-RR slope (0.211 ± 0.099 vs. 0.220 ± 0.085; *p = *0.44).

## Discussion

Evaluation of QTi in this large cohort (254 LQTS patients and 201 control individuals) indicated that gender specific cut-off values are preferred. Based on the ROC curves, we propose a cut-off of 430 ms in males and 445 ms in females. Using these cut-offs, we achieved a high diagnostic accuracy of 91% in the total population (91% in males and 92% in females), which was confirmed in our validation cohort of previously published LQTS patients. Furthermore, repeat holter recordings in 76 LQTS patients resulted in very similar values of QTi demonstrating replication of the findings and low intra-individual variability. The sensitivity of the test was somewhat lower in LQT1 patients, not related to the magnitude of the functional defect of the specific mutations. Similar reduced sensitivities in LQT1 have been observed with the brisk standing test, but not with the exercise test (4, 5). Also, QTi was not different in mutation carriers according to their effect on the amino acid sequence (missense or truncating) or location of the mutation.

Evaluation of QTi at RR intervals different from conventional 1,000 ms showed that starting from an unknown genotype, QTi at 1,000 ms was non-inferior to QT interval at any other RR interval. This was also true for patients with LQT2, but in LQT1 QTi measured at an RR interval of 900 ms in both genders and 800 ms in males performed significantly better to discriminate between genotype positive patients and controls compared to QTi at 1,000 ms. Therefore, it might be interesting to look at QTi values at faster heart rates in family members of LQT1 patients. This is also reflected in the fact that at fast heart rates (RR 600 ms) QTi was longer in LQT1 compared to LQT2, while at slow heart rates (from 60 BPM on) QTi was longer in LQT2 compared to LQT1.

Diagnostic accuracy of LQTS using a standard QTc derived from resting ECG is insufficient (2). However, since patients with so-called concealed LQTS (carrying a LQTS mutation with normal QTc at resting ECG) are nonetheless at increased risk of cardiac events, it is essential to quickly identify these at risk individuals. Several methods have been proposed to do so. Most of these rely on the pathologic prolongation of the QT interval in response to adrenergic stimulation. These include the adrenaline challenge, exercise test and the brisk standing test (3–5). Multiple potential advantages of using QTi derived from 24 h holter recordings are evident and might overcome issues that complicate correct diagnosis in LQTS.

First, it is common practice to correct the QT interval at a given heart rate to the QT interval you expect to have at an RR interval of 1,000 ms (60 BPM). This “corrected” value is also the value that is used to diagnose, and risk stratify LQTS. QT correction for heart rate is traditionally done using Bazett's formula (QTc = QT/RR^(1/2)) (14). This is problematic since it is well known that this formula overcorrects both at fast and slow heart rates because it assumes a non-physiological QT rate dependence (15). Indeed, this formula and other generalized formulae, are derived from QT-RR behaviour from small (Bazett's formula is based on the study of only 39 individuals) or larger groups (Framingham formula is based on over 5,000 individuals) of normal individuals (14, 16). However, it was shown that the QT-RR relationship exhibits important intersubject variability obscuring the use of a generalized formula (17). This intersubject variability is even more pronounced in LQTS, because the disease is characterized by altered QT rate dependence, especially in patients with LQT2 and LQT3 as was shown previously (18) and which is also evident from our data. Furthermore, the intrasubject stability of the QT-RR relation was reported to be high (17), which is also evident in our data since there was no difference in QT-RR slope in 76 LQTS patients with repeat recordings. The use of corrected QT calculation based on the patient's own QT-RR profile therefore is preferred over any generalized formula, like we showed previously for Bazett's formula (11).

Second, a standard ECG only evaluates 10 s of ECG. This is problematic since the QT interval has an important circadian variability (19). This was recently shown again in an elegant study by the group of Couderc, who plotted QTc on a 24 h clock (7). This variability is due to changes in heart rate, electrolyte disturbances, and autonomic modulation. Therefore, inclusion of 24 h of ECG recording in the evaluation of the QT interval will result in a more correct assessment of the QT interval.

Third, it is an easy to perform test that can already be calculated from implemented QT-RR modules in different commercial available holter software packages like those from Microport (Synescope as was used in this study) and GE (MARS).

Finally, a test with high diagnostic accuracy can aid in genetic variant interpretation. Indeed, classification of novel genetic variants often relies upon co-segregation analysis. Increasing the ability to correctly classify variant carriers or non-carriers as clinically affected by the disease increases the likelihood of classifying a genetic variant as either benign if co-segregation does not fit or (likely) pathogenic if sufficient numbers of gene carriers are affected (20).

## Study limitations

This study focused on patients and families with LQTS. The prevalence of the disease in these cohorts was 56%. Therefore, the diagnostic test results can only be used in the setting of familial LQTS. Since there is an overlap of QT between healthy individuals at the extreme end of the normal spectrum and LQTS patients, caution should be used when applying the proposed normal values to the general population.

There were only 13 patients with LQT3 included in the study. This precluded a detailed analysis of QTi and QT-RR slope in LQT3 patients. Likewise, only 5 LQTS patients in this cohort had a documented TdP or cardiac arrest. Since the low numbers, no evaluation of patients with these endpoints was performed.

Conventional 10 s ECG's were not available in the THEW database. Therefore, direct comparison of conventional QTc with QTi was not possible in this cohort. However our previous data already showed superiority of QTi over QTc (11).

Finally, QT-RR curvatures are subject specific and might be curvilinear rather than linear (21). In this study, calculation of QTi was based on linear regression of QT-RR data because of software limitations.

## Conclusions

Individualized QT interval (QTi) appeared to be a test with a very high diagnostic accuracy in a large cohort of LQTS mutation carriers. We propose modified cut-off values of 430 ms in males and 445 ms in females. Compared to traditional QT correction with generalized formulas like Bazett's formula that estimate or predict the QT interval at a heart rate of 60 BPM, QTi is a definite measurement that cancels out the need for QT correction. Our findings further demonstrate that QTi is a reliable tool to diagnose LQTS.

## Data Availability

The raw data supporting the conclusions of this article will be made available by the authors, without undue reservation.
