# Supplementary material for: Individualized QT interval (QTi) is a powerful diagnostic tool in long QT syndrome: results from a large validation study
Source: Front Cardiovasc Med. 2023 May 11;10:1097468. doi: 10.3389/fcvm.2023.1097468 (PMC10213876; doi:10.3389/fcvm.2023.1097468)
Supplement: Supplementary file 1 [file Table1.docx]

**SUPPLEMENTAL MATERIAL**

**Individualized QT interval (QTi) is a powerful diagnostic tool in Long QT Syndrome: results from a large validation study**

**Robyns T et al.**

**Table of Contents:**

Page 2: Type and location of mutation: methods and results

Page 3-5: Supplemental Table 1: Mutations included in the analyses (page 1-

Page 6: Supplemental Table 2: QTi at different RR intervals in male LQTS patients and control individuals (page

Page 7: Supplemental Table 3: QTi and QT-RR slope according to location of the mutation

Page 8: Supplemental Figure 1: ROC curve of QTi value at different RR intervals in LQTS patients and controls

Page 9: Supplemental Figure 2: ROC curve of QTi value at different RR intervals in LQT1 patients and controls

Page 10: Supplemental Figure 3: ROC curve of QTi value at different RR intervals in LQT2 patients and controls

Page 11: References supplemental data

***Type and location of mutation: methods***

Mutations were classified as missense, truncating (frameshift mutation or introduction of premature stop codon) or splice site mutation. In *KCNQ1*, 5 different mutation locations were defined: N-terminus (amino acid (AA) 1-121), transmembrane S1-S4 (AA 122-168 and 197-248), cytoplasmic loops (AA 169-196 and 249-261), pore (AA 262-348) and C-terminus (AA 349-676).^1^ In *KCNH2*, 6 different mutation locations were defined: Per-Arnt-Sim (PAS) domain (AA 41-70), PAS associated C-terminal domain (PAC, AA 92-144), remainder of N-terminus, transmembrane S1-S4 (AA 398-551), pore region (AA 552-657), C-terminus (AA 658-1159).^2^

**Type and location of mutation: results**

In LQT1, 148 holter recordings belonged to patients with a missense mutation, 4 with a truncating mutation and 7 with a splice site mutation. No significant differences were observed probably due to the low numbers. In LQT2, QTi in patients with a missense mutation (N=56; 492±35 ms) showed a trend (*p=*0.092) towards increased duration compared to patients with a truncating mutation (N=11, 470±23 ms) or splice site mutation (N=11, 476±36 ms). There were no statistical significant differences in QTi dependent upon the location of the mutation in both LQT1 and LQT2 (supplemental table 3). However, in both genotypes the longest QTi was observed in mutations that were located in the pore region of the channel.

***Supplemental table 1:*** *Mutations included in the analysis*

| **Gene** | **cDNA** | **Protein** | **ACMG-AMP** | **ExAC** | **Clinvar** | **N holters** | **N patients** |
| --- | --- | --- | --- | --- | --- | --- | --- |
| **LQT1** |  |  |  |  |  |  |  |
| *KCNQ1* | c.502G>C | p.(Gly168Arg) | P | 0/119588 | 1P | 7 | 4 |
| *KCNQ1* | c.520C>T | p.(Arg174Cys) | P | 1/119674 | 1P | 10 | 7 |
| *KCNQ1* | c.521G>A | p.(Arg174His) | LP | 1/119698 | 1LP | 11 | 8 |
| *KCNQ1* | c.536G>C | p.(Gly179Ala) | LP | 10/117790 | - | 4 | 2 |
| *KCNQ1* | c.569G>A | p.(Arg190Gln) | P | 0/119832 | 4P | 4 | 4 |
| *KCNQ1* | c.674C>T | p.(Ser225Leu) | P | 2/119708 | 1LP | 7 | 5 |
| *KCNQ1* | c.691C>T | p.(Arg231Cys) | P | 0/111544 | 1P,1LP | 3 | 2 |
| *KCNQ1* | c.728G>A | p.(Arg243His) | P | 0/115468 | - | 6 | 2 |
| *KCNQ1* | c.760G>A | p.(Val254Met) | P | 0/111368 | 3P, 1LP | 16 | 12 |
| *KCNQ1* | c.773A>C | p.(His258Pro) | P | 0/106426 | - | 1 | 1 |
| *KCNQ1* | c.780+2_780+20del |  | P | 0/98672 | - | 4 | 3 |
| *KCNQ1* | c.781G>A | p.(Glu261Lys) | P | 0/119292 | - | 5 | 4 |
| *KCNQ1* | c.805G>A | p.(Gly269Ser) | LP | 1/119460 | 1P | 1 | 1 |
| *KCNQ1* | c.806G>A | p.(Gly269Asp) | P | 0/119460 | 1P | 3 | 3 |
| *KCNQ1* | c.830C>T | p.(Ser277Leu) | P | 0/119448 | 2P,1LP | 3 | 2 |
| *KCNQ1* | c.830C>G | p.(Ser277Trp) | LP | 0/119448 | 1P | 5 | 2 |
| *KCNQ1* | c.922-2A>G |  | P | 0/120902 | - | 3 | 1 |
| *KCNQ1* | c.926C>G | p.(Thr309Arg) | LP | 0/120978 | - | 3 | 1 |
| *KCNQ1* | c.940G>A | p.(Gly314Ser) | P | 0/121048 | 1P | 15 | 8 |
| *KCNQ1* | c.944A>C | p.(Tyr315Ser) | P | 0/121060 | - | 13 | 10 |
| *KCNQ1* | c.947G>A | p.(Gly316Glu) | P | 0/121092 | - | 1 | 1 |
| *KCNQ1* | c.958C>G | p.(Pro320Ala) | P | 0/121120 | - | 15 | 9 |
| *KCNQ1* | c.973G>A | p.(Gly325Arg) | P | 0/121142 | 1LP, 1P | 7 | 7 |
| *KCNQ1* | c.1031C>T | p.(Ala344Val) | LP | 0/120972 | 1P | 8 | 7 |
| *KCNQ1* | c.1032G>A | p.(Ala344Ala) | P | 0/120864 | 3P | 7 | 4 |
| *KCNQ1* | c.1096C>T | p.(Arg366Trp) | LP | 0/120884 | 1P, 1LP | 1 | 1 |
| *KCNQ1* | c.1111G>A | p.(Ala371Thr) | P | 0/120772 | - | 1 | 1 |
| *KCNQ1* | c.1265dup | p.(Phe423Valfs*40) | LP | 1/119632 | - | 1 | 1 |
| **Gene** | **cDNA** | **Protein** | **ACMG-AMP** | **ExAC** | **Clinvar** | **N holters** | **N patients** |
| *KCNQ1* | c.1343del | p.(Pro448Glnfs*18) | P | 1/118590 | 1P | 1 | 1 |
| *KCNQ1* | c.1343dup | p.(Glu449Argfs*14) | P | 0/118590 | 1P | 1 | 1 |
| *KCNQ1* | c.1615C>T | p.(Arg539Trp) | P | 0/25830 | 2P | 16 | 7 |
| *KCNQ1* | c.1663C>T | p.(Arg555Cys) | P | 1/27888 | 2P | 33 | 32 |
| *KCNQ1* | c.1664G>A | p.(Arg555His) | LP | 0/27888 | 1P | 7 | 4 |
| *KCNQ1* | c.1700T>C | p.(Ile567Thr) | LP | 0/119994 | 2P | 1 | 1 |
| *KCNQ1* | c.1760C>G | p.(Thr587Arg) | LP | 0/120684 | - | 1 | 1 |
| *KCNQ1* | c.1772G>A | p.(Arg591His) | P | 0/120686 | - | 2 | 2 |
| *KCNQ1* | Unknown deletion 19 nucleotides exon 5# |  | LP |  |  | 2 | 1 |
| **LQT2** |  |  |  |  |  |  |  |
| *KCNH2* | c.131G>T | p.(Cys44Phe) | P | 0/94696 | - | 3 | 3 |
| *KCNH2* | c.145T>C | p.(Cys49Arg) | LP | 0/94696 | - | 1 | 1 |
| *KCNH2* | c.172G>A | p.(Glu58Lys) | P | 0/82810 | - | 8 | 4 |
| *KCNH2* | c.298C>G | p.(Arg100Gly) | P | 0/39626 | 1P | 2 | 1 |
| *KCNH2* | c.340C>T | p.(Pro114Ser) | LP | 0/121230 | - | 15 | 3 |
| *KCNH2* | c.371T>G | p.(Met124Arg) | P | 0/121320 | - | 3 | 1 |
| *KCNH2* | c.1008_1009insT | p.(Thr337Tyrfs*19) | P | 0/119212 | - | 3 | 2 |
| *KCNH2* | c.1129-1G>C |  | LP | 0/119244 | - | 9 | 9 |
| *KCNH2* | c.1205A>G | p.(His402Arg) | LP | 0/121346 | - | 4 | 2 |
| *KCNH2* | c.1389C>G | p.(Phe463Leu) | P | 0/121404 | - | 1 | 1 |
| *KCNH2* | c.1424A>G | p.(Tyr475Cys) | LP | 0/121400 | - | 3 | 1 |
| *KCNH2* | c.1501G>A | p.(Asp501Asn) | P | 0/121316 | 1P | 4 | 3 |
| *KCNH2* | c.1681G>A | p.(Ala561Thr) | P | 0/121016 | 1P | 2 | 2 |
| *KCNH2* | c.1704G>T | p.(Trp568Cys) | P | 0/121150 | - | 5 | 3 |
| *KCNH2* | c.1724A>G | p.(Glu575Gly) | P | 0/121032 | - | 1 | 1 |
| *KCNH2* | c.1744C>T | p.(Arg582Cys) | P | 0/121250 | 1P | 8 | 4 |
| *KCNH2* | c.1786C>A | p.(Pro596Thr) | LP | 0/121300 | - | 2 | 1 |
| *KCNH2* | c.1801G>A | p.(Gly601Ser) | P | 0/121300 | 2P | 1 | 1 |
| *KCNH2* | c.1810G>A | p.(Gly604Ser) | P | 0/121308 | 2P | 3 | 3 |
| **Gene** | **cDNA** | **Protein** | **ACMG-AMP** | **ExAC** | **Clinvar** | **N holters** | **N patients** |
| *KCNH2* | c.1825G>A | p.(Asp609Asn) | P | 0/121326 | - | 3 | 3 |
| *KCNH2* | c.1838C>T | p.(Thr613Met) | P | 0/121320 | 1P, 1LP | 6 | 5 |
| *KCNH2* | c.1862G>A | p.(Ser621Asn) | P | 0/121326 | - | 1 | 1 |
| *KCNH2* | c.1898A>G | p.(Asn633Ser) | P | 0/121314 | 1P | 8 | 3 |
| *KCNH2* | c.1900A>G | p.(Thr634Ala) | LP | 0/121314 | 1P | 2 | 2 |
| *KCNH2* | c.2231G>C | p.(Arg744Pro) | LP | 0/118654 | - | 2 | 2 |
| *KCNH2* | c.2399-2A>G |  | P | 0/112916 | - | 11 | 2 |
| *KCNH2* | c.2453C>T | p.(Ser818Leu) | P | 1/121198 | 1P | 4 | 2 |
| *KCNH2* | c.2464G>A | p.(Val822Met) | P | 0/121280 | 1P | 3 | 3 |
| *KCNH2* | c.3107dup | p.(Asp1037Argfs*82) | P | 0/11796 | 1P | 4 | 2 |
| *KCNH2* | c.3251del | p.(Pro1084Argfs*171) | P | 0/116862 | - | 3 | 3 |
| *KCNH2* | Unknown delins of 13 nucleotides at position c.1033# |  | LP | 0/114876 |  | 2 | 1 |
| *KCNH2* |  | Unknown stop at p.644# | LP |  |  | 4 | 3 |
| **LQT3** |  |  |  |  |  |  |  |
| *SCN5A* | c.1007C>T | p.(Pro336Leu) | LP | 0/118252 | - | 3 | 1 |
| *SCN5A* | c.5329G>A | p.(Val1777Met) | LP | 3/121080 | 1LP | 10 | 3 |
| *SCN5A* | c.5350G>A | p.(Glu1784Lys) | P | 0/121022 | 5P | 3 | 1 |
| *SCN5A* | c.5369A>G | p.(Asp1790Gly) | P | 0/120894 | 1P | 17 | 8 |

*# = These mutations could not be reconstructed based on the details in the database. However, since they cause either introduction of a stop codon or a frameshift they were classified as likely pathogenic and retained for further analysis; ACMG-AMP refers to the criteria for interpretation of sequence variants according the American College of Medical Genetics and Genomics and the Association for Molecular Pathology; EXaC = frequency of the variant in the Exome aggregation consortium; Clinvar refers to classification of the variant according the Clinvar database; P = pathogenic variant; LP = likely pathogenic variant; N = number.*

***Supplemental table 2:*** *QTi at different RR intervals in male LQTS patients and control individuals*

|  | Males | | | Females | | |  |
| --- | --- | --- | --- | --- | --- | --- | --- |
|  | Controls | LQTS | *p*-value | Controls | LQTS | *p*-value |  |
| Number | 102 | 115 |  | 99 | 139 |  |  |
| QTi (ms) | 390 ± 19 | 465 ± 36 | <0.001 | 409 ± 21 | 475 ± 34 | <0.001 |  |
| QTi 600 (ms) | 332 ± 16 | 380 ± 27 | <0.001 | 333 ± 18 | 387 ± 34 | <0.001 |  |
| QTi 700 (ms) | 346 ± 16 | 401 ± 25 | <0.001 | 352 ± 17 | 409 ± 30 | <0.001 |  |
| QTi 800 (ms) | 361 ± 16 | 422 ± 26 | <0.001 | 371 ± 17 | 491 ± 29 | <0.001 |  |
| QTi 900 (ms) | 376 ± 17 | 443 ± 30 | <0.001 | 390 ± 18 | 453 ±31 | <0.001 |  |
| QTi 1100 (ms) | 405 ± 21 | 486 ± 43 | <0.001 | 428 ± 24 | 497 ± 40 | <0.001 |  |
| QTi 1200 (ms) | 420 ± 23 | 507 ± 51 | <0.001 | 447 ± 27 | 519 ± 46 | <0.001 |  |
| QTi 1300 (ms) | 434 ± 25 | 528 ± 59 | <0.001 | 466 ± 31 | 541 ± 53 | <0.001 |  |
| QTi 1400 (ms) | 449 ± 28 | 549 ± 68 | <0.001 | 485 ± 35 | 563 ± 61 | <0.001 |  |

*QTi = Individualized QT correction*

***Supplemental table 3:*** *QTi and QT-RR slope according to location of the mutation*

|  | N | QTi | QT-RR slope |
| --- | --- | --- | --- |
| **LQT1** |  |  |  |
| Transmembrane S1-S4 | 13 | 460±31 | 0.171±0.056 |
| C-loop | 38 | 460±28 | 0.151±0.080 |
| Pore | 52 | 469±32 | 0.191±0.080 |
| C-terminus | 49 | 453±32 | 0.192±0.062 |
| *p*-value |  | 0.089 | 0.033* |
| **LQT2** |  |  |  |
| PAS | 8 | 480±17 | 0.223±0.043 |
| PAC | 5 | 484±37 | 0.231±0.114 |
| Transmembrane S1-S4 | 7 | 494±45 | 0.290±0.059 |
| Pore | 29 | 502±35 | 0.309±0.091 |
| C-Terminus | 7 | 465±30 | 0.267±0.043 |
| *p*-value |  | 0.107 | 0.059 |

*LQT1 = long QT type 1; LQT2 = long QT type 2; C-loop = cytoplasmic loop; PAS = Per-Arnt-Sim domain; PAC = PAS associated C-terminal domain; QTi = Individualized QT correction; * Post hoc testing shows significant difference between C-loop and C-terminus*


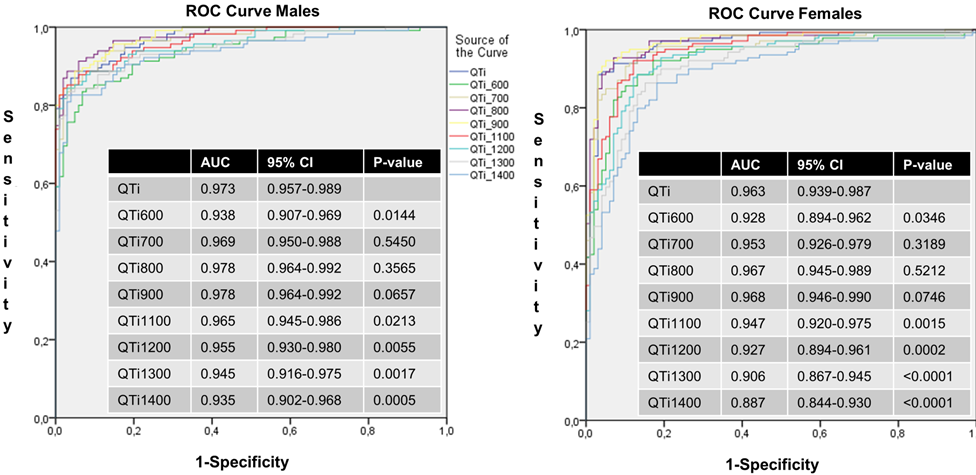


***Supplemental figure 1 :*** *ROC curve of QTi value at different RR intervals in male LQTS patients versus male controls (left panel) and in female LQTS patients and female controls (right panel). p-value indicates the p-value of the ROC curve of the specific QTi compared to QTi at 1000 ms (denoted as QTi in the table).*


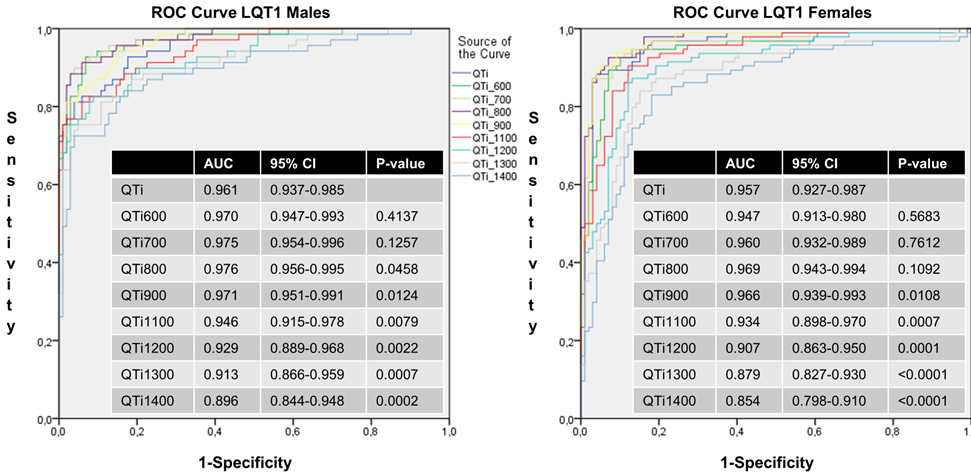


***Supplemental figure 2:*** *ROC curve of QTi value at different RR intervals in male LQT1 patients versus male controls (left panel) and in female LQT1 patients versus female controls (right panel). p-value indicates the p-value of the ROC curve of the specific QTi compared to QTi at 1000 ms (denoted as QTi in the table).*


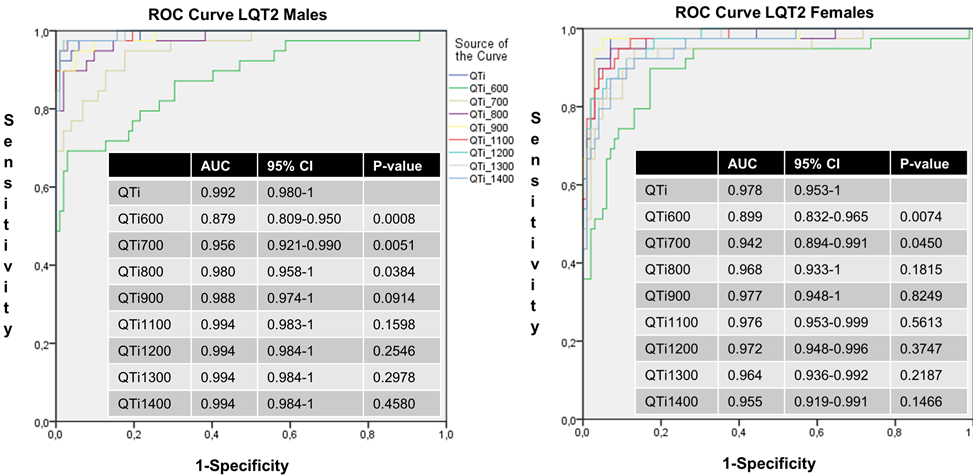


***Supplemental figure 3 :*** *ROC curve of QTi value at different RR intervals in male LQT2 patients versus male controls (left panel) and in female LQT2 patients and female controls (right panel). p-value indicates the p-value of the ROC curve of the specific QTi compared to QTi at 1000 ms (denoted as QTi in the table).*

**References supplemental data**

1. Wu J, Ding WG, Horie M. Molecular pathogenesis of long QT syndrome type 1. J Arrhythm 2016;**32**(5):381-388.

2. Shimizu W, Moss AJ, Wilde AA, Towbin JA, Ackerman MJ, January CT, Tester DJ, Zareba W, Robinson JL, Qi M, Vincent GM, Kaufman ES, Hofman N, Noda T, Kamakura S, Miyamoto Y, Shah S, Amin V, Goldenberg I, Andrews ML, McNitt S. Genotype-phenotype aspects of type 2 long QT syndrome. J Am Coll Cardiol 2009;**54**(22):2052-62.
